# Bedside determination of microcirculatory oxygen delivery and uptake: a prospective observational clinical study for proof of principle

**DOI:** 10.1038/s41598-021-03922-4

**Published:** 2021-12-31

**Authors:** Timo Sturm, Julia Leiblein, Christoph Clauß, Enno Erles, Manfred Thiel

**Affiliations:** 1 Department of Anaesthesiology and Surgical Intensive Care Medicine, University Medical Centre Mannheim, Medical Faculty Mannheim, Heidelberg University, Theodor-Kutzer-Ufer 1-3, 68167 Mannheim, Germany; 2 TRACC-Group (Translational Research in Anaesthesiology and Critical Care), Mannheim, Germany

## Abstract

Assessment of microcirculatory functional capacity is considered to be of prime importance for therapy guidance and outcome prediction in critically ill intensive care patients. Here, we show determination of skin microcirculatory oxygen delivery and consumption rates to be a feasible approach at the patient’s bedside. Real time laser-doppler flowmetry (LDF) and white light spectrophotometry (WLS) were used for assessment of thenar skin microperfusion, regional Hb and postcapillary venous oxygen saturation before and after forearm ischemia. Adapted Fick’s principle equations allowed for calculation of microcirculatory oxygen delivery and uptake. Patient groups with expected different microcirculatory status were compared [control (n = 20), sepsis-1/2 definition criteria identified SIRS (n = 10) and septic shock patients (n = 20), and the latter group further classified according to sepsis-3 definition criteria in sepsis (n = 10) and septic shock (n = 10)], respectively. In otherwise healthy controls, microcirculatory oxygen delivery and uptake approximately doubled after ischemia with maximum values (mDO2max and mVO2max) significantly lower in SIRS or septic patient groups, respectively. Scatter plots of mVO2max and mDO2max values defined a region of unphysiological low values not observed in control but in critically ill patients with the percentage of dots within this region being highest in septic shock patients. LDF and WLS combined with vasoocclusive testing reveals significant differences in microcirculatory oxygen delivery and uptake capacity between control and critically ill patients. As a clinically feasible technique for bedside determination of microcirculatory oxygen delivery and uptake, LDF and WLS combined with vasoocclusive testing holds promise for monitoring of disease progression and/or guidance of therapy at the microcirculatory level to be tested in further clinical trials.

**ClinicalTrials.gov**: NCT01530932.

## Introduction

Major advances have been made in hemodynamic monitoring of critical ill patients in the last decades. In the 1970s, intravascular pressure monitoring was introduced and later pulmonary artery thermodilution catheters revolutionised intensive care monitoring^1^. Flow-based parameters extended the spectrum of clinical monitoring techniques allowing for bedside calculation of systemic vascular resistance, global oxygen delivery (DO2) and uptake (VO2), considered to be hemodynamic diagnostic milestones. In the early 2000s, echocardiography of the beating heart and valvular function as well as ultrasound assessment of vascular filling became clinical routine. The use of pulmonary artery catheters was more and more restricted by a lack of evidence of clinical usefulness^2,3^. Transpulmonary cardiac output monitoring became popular und global oxygen consumption / delivery balance was estimated by central venous oxygen saturation^4^. All these approaches focus on the macrocirculatory level and hence do not directly provide information of what happens at the microcirculatory and cellular level. Any restriction in oxygen delivery to cells, however, will promote cellular dysfunction^1^, organ failure^5,6^ and will end up in the break-down of biological barrier functions leading to a loss in protection against bacteria, thereby increasing rates of infections and associated secondary problems^7^. Neuroendocrine stress reactions and therapeutically infused vasopressors induce blood flow redistribution and affect peripheral organs like the skin^8^ and the digestive tract^9^, known to be low flow areas in critical illness. Thus, in the present study we set out to investigate the technical feasibility and clinical relevance of skin microcirculatory oxygen balance measurements. To this end, preoperative otherwise healthy control patients, patients suffering from non-infectious systemic inflammation after trauma or major surgery, and septic shock patients were tested for differences in microcirculatory flow dependent oxygen delivery and oxygen consumption rates upon vasooclusive testing.

## Methods

The Ethics Commission II of the Medical Faculty Mannheim approved the study (2011-411 M-MA). All methods were performed in accordance with relevant guidelines/regulations including the Declaration of Helsinkie. All patients or their relatives, if patients’ mental status was not adequate to make this decision, gave informed consent. Upon their recovery, previously not self-determined patients had the opportunity to withdraw their consent to participate in this study. Study planning and reporting were adapted to the STROBE statement recommendations (http://www.strobe-statement.org/). Three cohorts were enrolled from August 2012 to April 2014: non-infected control patients listed for elective minor surgery, patients suffering from non-infectious systemic inflammatory response syndrome (SIRS) following major trauma or surgery and septic shock patients as defined by sepsis-1/2 definition criteria^10^, respectively. Sepsis-3 definition criteria^11^ were retrospectively applied to sepsis-1/2 defined septic shock patients to allow for patient classification into sepsis-3 and septic shock-3 patient groups, respectively. Data acquisition was performed at recovery rooms and surgical intensive care units (ICU) of the University Medical Centre Mannheim. The septic patient groups had prolonged microcirculatory monitoring until day four of ICU treatment and were additionally analysed to identify other microcirculatory targets potentially suitable for guidance of hemodynamic therapy^12^. All data presented were taken from patients as soon as possible after onset of syndromes usually within the next 24 h.

### Patients

Control group comprised age- and sex-matched patients in order to avoid bias by comparing values of healthy young volunteers to those of elderly ICU collectives with relevant medical history. Control group patients were scheduled for elective minor surgery without any anamnestic or laboratory indicators of systemic inflammation (subjective feeling of malaise, absence of fever and abnormal white blood cell count or elevated C-reactive protein plasma concentration).

Group of SIRS patients had encountered major trauma (n = 3), laparoscopic-thoracoscopic esophagectomy (n = 2) or open cystectomy (n = 5). Among all SIRS patients, mechanical ventilation was required in two polytrauma patients. Infusion of low-dose norepinephrine was required in two polytrauma patients and two patients after eosophagectomy. Patients were hemodynamically stable as there was no need for invasive flow-based cardiovascular monitoring. None of them developed acute kidney injury. There were no clinical signs or microbiological evidence for bacterial infection to account for systemic inflammation. As juged by CRP plasma levels, intensity of systemic inflammation was mild (see Table 1).

**Table 1:** Clinical characteristics of control and patient groups; SAPS II, Simplified Acute Physiology Score II; SOFA, Sequential Organ Failure Assessment score; estimated mortality, SAPS II scoring based mortality prediction; WBC, white blood cell count; CRP, plasma concentration of C-reactive protein; PCT, plasma concentration of procalcitonin; SaO_2_, arterial blood oxygen saturation; SpO_2_, pulse oximetry saturation; ScvO_2_, central venous oxygen saturation; HR, heart rate; MAP, mean arterial pressure; data are presented as mean ± SD (median); & or * indicate “as compared to control”, respectively; § indicates “as compared to SIRS”; # indicates “as compared to sepsis-3”; a *p*-value < 0.05 was considered as statistically significant.

Clinical data Mean ± SD (median)	Control n = 20	SIRS n = 10	Septic shock-1/2 n = 20	Sepsis-3 n = 10	Septic shock-3 n = 10
Gender male (%)	10 (50)	8 (80)	10 (50)	5 (50)	5 (50)
Age–years	63.7 ± 12.0 (66.5)	60.4 ± 18.7 (62.5)	61.8 ± 16.8 (68)	51.1 ± 17.0 (45)*	72.4 ± 7.0 (74.0)^#^
**Severity of illness**					
SAPS II	–	26.6 ± 11.9 (22.5)	59.2 ± 14.9 (58.0)^§^	48.3 ± 11.2 (51.0)	70.1 ± 9.0 (73.5)^#^
SOFA	–	4.8 ± 3.0 (6.0)	13.3 ± 3.1 (14.0)^§^	12.2 ± 3.3 (12.5)	14.4 ± 2.5 (14.5)
Estimated mortality (%)	–	12.9 ± 3.9 (9.9)	59.9 ± 24.1 (55.0)^§^	52.2 ± 26.0 (50)	67.5 ± 20.2 (67.5)
**Lab data**					
pH	7.38 ± 0.03 (7.38)	7.40 ± 0.04 (7.40)	7.34 ± 0.12 (7.33)	7.38 ± 0.1 (7.36)	7.28 ± 0.2 (7.30)*
Haemoglobin (g/dl)	13.8 ± 1.4 (14.0)	11.7 ± 1.7 (12.3)^&^	10.4 ± 2.0 (9.9)^&^	10.0 ± 2.0 (9.5)*	10.9 ± 2.1 (10.0)*
Platelets count (n/nl)	245 ± 66 (259)	197 ± 57 (210)	173 ± 111 (172)	212 ± 108 (196)	166 ± 116 (159)*
Quick value (%)	103 ± 13 (103)	77 ± 13 (75)^&^	66 ± 21 (63)^&^	70 ± 24 (72)*	63 ± 17 (61)*
WBC (n/nl)	7.2 ± 2.1 (7.1)	15.5 ± 3.0 (14.2)^&^	15.0 ± 11.0 (12.5)^&^	14.8 ± 11.9 (11.8)*	15.0 ± 12.5 (10.6)*
CRP (mg/l)	6.6 ± 7.0 (2.9)	33.1 ± 53.5 (7.7)	221.7 ± 119.4 (247)^&,§^	239 ± 126 (266)*	204 ± 116 (230)*
PCT (μg/l)	–	1.8 ± 3.3 (0.6)	47.5 ± 91.7 (9.6)	50.8 ± 122.0 (4.1)	44.2 ± 60.5 (23.0)
SaO_2_ / SpO_2_ (%)	95.8 ± 1.6 (96.0)	97.9 ± 1.9 (98.5)	92.9 ± 4.4 (93.5)^&,§^	94.1 ± 3.5 (94.0)	91.7 ± 5.1 (92.5)*
ScvO_2_ (%)	–	71.2 ± 7.8 (74)	73.0 ± 4.9 (74)	73.3 ± 5.1 (74.0)	72.8 ± 5.0 (74.0)
Lactate (mmol/l)	1.3 ± 0.4 (1.3)	2.4 ± 1.9 (2.1)	3.2 ± 2.3 (2.3)^&^	1.5 ± 0.4 (1.6)	4.9 ± 2.3 (4.8)*^,#^
**Hemodynamic data**					
Heart rate (/min)	74.3 ± 8.1 (72.5)	92.4 ± 20.1 (90.0)^&^	105.3 ± 18.6 (103.5)^&,§^	100.2 ± 18.6 (103.5)*	110.3 ± 18.2 (103.5)*
MAP (mmHg)	96.1 ± 12.6 (94.0)	88.3 ± 11.9 (89.5)	73.6 ± 9.7 (71.0)^&,§^	76.5 ± 11.4 (73.0)*	70.7 ± 7.1 (69.0)*
Cardiac index (l/min/m^2^)	–	–	3.95 ± 1.28 (4.0)	4.26 ± 1.23 (4.1)	3.63 ± 1.29 (3.48)
Norepinephrine (μg/kg/min)	–	0.24 ± 0.19 (0.19)	0.59 ± 0.50 (0.42)	0.29 ± 0.27 (0.21)	0.84 ± 0.60 (0.88)^#^

Sepsis and septic shock was caused by bacterial infection due to abdominal whole organ perforation, intestinal ischemia or peritonitis in 10 cases or due to pneumonia and ARDS in the other 10 cases. Bacterial spectrum was typical for abdominal or pulmonary source of infection comprising gram-negative *Enterobacteriacea species* or gram-positive *Streptococci species*, respectively. No bacteria could be detected in four cases. All patients were intubated and mechanically ventilated. Cardiovascular function monitoring was instituted as described below.

Since classic SIRS definition^10^ is hardly applicable to mechanically ventilated, vasopressor supported and temperature controlled ICU populations, criteria for heart rate and tachypnea were adapted in conformity with methodology recently published^13^.

### Assessment of severity of critical illness and mortality prediction

Severity of disease and mortality prediction was assessed by SOFA^14^ and SAPS II^15^, respectively.

### Clinical data measurements

Hemodynamic parameters (arterial blood pressure, central venous pressure, heart rate) were recorded by both IntelliVue MP 70 (PHILIPS Healthcare, Germany) monitoring for ICU patients and Dash 3000 (GE Medical, Great Britain) monitoring for controls. Cardiac index was monitored every eight hours as part of routine clinical care and before vasoocclusive testing. For this, patients had a femoral or axillary thermistor-tipped arterial catheter (5 F/20 cm or 4F/16 cm, PULSION, Germany), and measurements were based on the transpulmonary thermodilution technique (PiCCO®, PULSION, Germany)^16^. Blood gas testing was processed by ABL 800 flex (RADIOMETER, Denmark) after blood sampling by venous puncture in control patients and collection from arterial and venous catheters in SIRS and septic shock collectives. Anamnestic and laboratory datasets were extracted from the local patient data management and hospital information system (IntelliSpace Critical Care and Anesthesia, PHILIPS Healthcare, Germany; IS-H med, SAP-Industry Solution, Germany).

### Microcirculatory measurements

**Figure 1:**
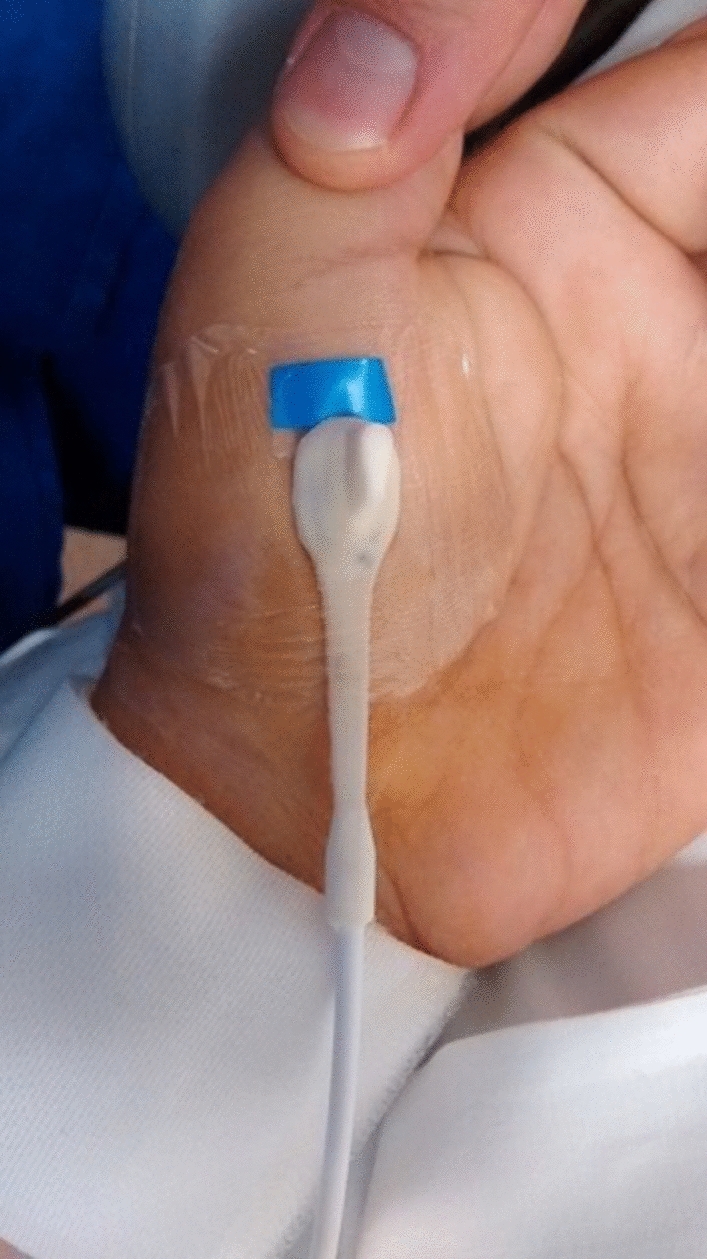
Laser-doppler flowmeter/white light spectrophotometry skin probe (LEA medical technology, Giessen, Germany) placing and fixation at thenar eminence.

A non-invasive microcirculatory monitoring device (O2C, LEA medical technology, Germany), which combines two optical methods, laser-doppler flowmetry (LDF) and white light spectrophotometry (WLS), was used for assessment of microperfusion (MBF, microcirculatory blood flow) and postcapillary venous oxygen saturation (SvcO2)^17–19^. A forearm vasoocclusive testing (VOT)-manoeuvre was performed by induction of ischemia–reperfusion using an inflatable tourniquet put to the forearm. The tourniquet was inflated up to 250 mm Hg, pressure kept for 3 min and rapidly relieved thereafter^20–22^. A flat light probe with two millimetre of separation between emitting and detecting area (LF2 probe) was gently fixed on the skin of the thenar with transparent double-sided adhesive tape (Fig. 1). The laser-doppler principle was applied for MBF recording based on the change in frequency of continuously transmitted monochromatic light at a wavelength of 830 nm (maximum internal power < 30 mW), emitted through a fibreoptic light guide. Flow of red blood cells in microvessels induce a frequency shift and the backscattered signal is processed by power spectral analyses, which allows for calculating the mean velocity and mean blood flow of moving erythrocytes. Quantitative assessment of the returning signals leads to relative MBF expressed in arbitrary units (AU) for about one cubic millimetre of tissue volume. In this particular setting it was detected at a depth of two millimetres under the skin surface of the thenar eminence.

WLS was used for detection of oxygen saturation. This is a technology, in which polychromatic light (wavelength range of 500–630 nm) is emitted into the tissue where it is consecutively scattered and absorbed, mainly by haemoglobin. Desoxygenated haemoglobin shows peak absorption at 556 nm. Oxygen-rich blood exhibits two absorption peaks, one above and one below this value. The change in measured absorption spectrum is used for the assessment of haemoglobin’s oxygen saturation^18^. The cumulated amount of absorption allows calculation of regional haemoglobin concentration (rHb). Postcapillary venules represent the main blood storage of the microcirculation, about 70–85% of haemoglobin is located in this area^17,23^, hence WLS measurements mainly reflect postcapillary venous oxygen saturation (SvcO2). Vessels larger than postcapillary venules do not contribute to signals as light is completely absorbed by vessels with diameters exceeding 100 µm^23^. MBF and SvcO2 were detected 40 times a minute that allows detailed registration of parameters under baseline conditions, in the ischemic period and during reperfusion. After two minutes of stabilisation, values collected in the next two minutes were averaged and mean value considered as baseline. Maximum values (MBFmax, SvcO2max) were collected during reactive hyperaemia phase after cuff deflation. Microcirculatory oxygen delivery (mDO2) and uptake (mVO2) were calculated in analogy to macrocirculatory mathematical derivations based on the Fick´s principle. For explanation, calculation of global oxygen delivery and consumption at the macrocirculatory level will first be briefly summarized as follows:

1 $${\text{DO}}_{2} = {\text{CO}} \times {\text{CaO}}_{2}$$

2 $${\text{VO}}_{2} = {\text{CO}} \times \, \left( {{\text{CaO}}_{2} - {\text{CvO}}_{2} } \right)$$

DO2 represents global oxygen delivery and VO2 global oxygen consumption. CO represents cardiac output, CaO2 and CvO2 arterial and venous oxygen content, respectively.

3 $${\text{CaO}}_{2} = {\text{Hb}} \times \, 1.39 \, \times {\text{SaO}}_{2} + \, 0.003 \, \times {\text{PaO}}_{2}$$

4 $${\text{CvO}}_{2} = {\text{Hb}} \times \, 1.39 \times {\text{SvO}}_{2} + 0.003 \, \times {\text{PvO}}_{2}$$

Hb indicates haemoglobin concentration, SaO2 arterial oxygen saturation and PaO2 arterial oxygen partial pressure. SvO_2_ represents mixed venous oxygen saturation. By combination of Eqs. (1with 3 and 2 with 4) DO_2_ and VO_2_ calculate to.

$${\text{DO}}_{2} = {\text{ CO}} \times ({\text{Hb}} \times 1.39 \times {\text{ SaO}}_{2} + 0.003 \times {\text{ PaO}}_{2} )$$

$${\text{VO}}_{2} = {\text{CO}} \times [{\text{Hb}} \times 1.39 \times ({\text{SaO}}_{2} - {\text{ SvO}}_{2} ) + 0.003 \times ({\text{PaO}}_{2} - {\text{ PvO}}_{2} )]$$

The fraction of physically dissolved oxygen is about a hundreth of the total quantity in standard clinical settings and hence can be neglected in practical applications.

$${\text{DO}}_{2} = {\text{CO}} \times {\text{Hb}} \times 1.39 \times {\text{SaO}}_{2}$$

$${\text{VO}}_{2} = {\text{CO}} \times {\text{Hb}} \times \, 1.39 \times \, ({\text{SaO}}_{2} - {\text{SvO}}_{2} )$$

These equations were adapted to the microcirculatory setting. After considering methodical particularities of unit-free MBF and rHb values there appears no need to take into account any longer the correction factor for oxygen binding capacity of haemoglobin. Thus, relatively simple equations result to calculate microcirculatory oxygen delivery and consumption:

$${\text{mDO}}_{2} = {\text{MBF}} \times {\text{rHb}} \times {\text{SaO}}_{2}$$

$${\text{mVO}}_{2} = {\text{MBF}} \times {\text{rHb}} \times \, ({\text{SaO}}_{2} - {\text{SvcO}}_{2} )$$

mDO2 represents microcirculatory oxygen delivery and mVO2 microcirculatory oxygen uptake, rHb regional haemoglobin concentration and SvcO2 postcapillary venous oxygen saturation.

Finally, SaO2 was replaced by pulse oximetry saturation (SpO2) for control patients to avoid unpleasant arterial punctures. In order to ensure easy to handle datasets, all mDO2 and mVO2 results were divided by one thousand.

### Statistical analyses

O2CevaTime-Software 20.2.0 (LEA medical technology, Giessen, Germany) and IBM SPSS Statistics 27 (IBM Corporation, Armonk, NY 10,504, USA) were used for data analyses. Chi-Square-tests and independent t-test or oneway ANOVA and post hoc LSD testing were used for intergroup comparison, including controls and sepsis-1/2—or sepsis-3 defined patient groups, respectively. Sepsis-1/2—and sepsis-3 defined groups were not compared as this was not the aim of the study. In fact, the study’s focus was search for an association between microcirculatory variables and states of increasing illness severity identified by both the still used sepsis-1/2^24,25^—and the more recently introduced sepsis-3 definition criteria^11^ to allow for proof of principle of the tested microcirculatory assessment method. We obtained Pearson correlation coefficients for clinical and hemodynamic parameters. Simple linear regression was used to estimate the line slope for graphical assessment of the association of selected parameters. For all analyses a p-value less than 0.05 (two-tailed) was considered statistically significant. Level of statistical significance was adjusted according to Bonferroni in case of multiple testing for pearson correlation.

### Ethics approval

This study protocol was approved by the Ethics Commission II of Medical Faculty Mannheim (2011-411 M-MA).

### Consent to participate

Written informed consent was obtained from each study participant or their next of kin upon their initial admission to the Intensive Care Unit.

## Results

A total of 50 patients in 5 groups ((control (n = 20), sepsis-1/2 defined SIRS (SIRS, n = 10) and sepsis-1/2 defined septic shock (septic shock-1/2, n = 20), and the latter group further classified by sepsis-3 criteria into sepsis (sepsis-3, n = 10) and septic shock (septic shock-3, n = 10)) were enrolled, VOT-manoeuvre was applied and associated microcirculatory analyses were performed.

### Clinical characteristics

Patients’ baseline characteristics are presented in Table 1. As compared to age- and sex matched control almost all parameters were increasingly altered with increasing illness severity reaching maximum changes in septic shock groups. Accordingly, SAPS II, SOFA scores and SAPS II based predicted mortality were significantly higher in septic shock-1/2 patients as compared with patients suffering from SIRS, with the same being true for the elevation of C-reactive protein, reflective of the degree of systemic inflammation. As compared to sepsis-3 patients, septic shock-3 patients were older and exhibited significantly higher SAPS II, lactate and norepinephrine infusion rates.

### Microcirculatory oxygen delivery and uptake

**Figure 2:**
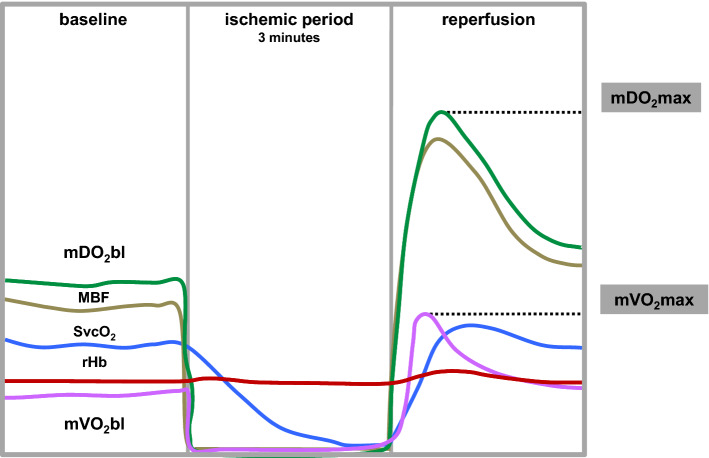
Schematic illustration of vasoocclusive testing: baseline detection, ischemic period and reperfusion after arterial inflow stop and release by cuff inflation and deflation, respectively; mDO2, microcirculatory oxygen delivery; mVO2, microcirculatory oxygen uptake; mDO2 and mVO2 were calculated as described in methods from continuously monitored variables MBF, rHb, SaO_2_ and SvcO_2_. MBF, microcirculatory blood flow; rHb, regional haemoglobin; SaO_2_, arterial blood oxygen saturation; SvcO2, postcapillary venous oxygen saturation.

The time course of measurement of microcirculatory parameters in response to VOT is illustrated in Fig. 2. Postcapillary venous oxygen saturation (SvcO2), regional haemoglobin (rHb) and microcirculatory blood flow (MBF) trend lines were directly recorded and mDO2 and mVO2 lines were calculated according to the formulas given in methods section. Baseline and peak values are given for the different groups in Table 2.

**Table 2:** Microcirculatory oxygen delivery and uptake by control and patient groups; mDO2, microcirculatory oxygen delivery; mVO2, microcirculatory oxygen uptake; MBF, Microcirculatory blood flow; rHb, regional haemoblobin; SaO_2_, arterial blood oxygen saturation, SpO_2_, pulse oximetry saturation; SvcO2, postcapillary venous oxygen saturation; mExO2, microcirculatory oxygen extraction rate; bl refers to baseline; max refers to maximum values determined upon reperfusion; data are presented as mean ± SD (median); & or * indicate “as compared to control”, respectively; § indicates “as compared to SIRS”; # indicates “as compared to sepsis-3”; a *p*-value < 0.05 was considered as statistically significant.

Microcirculatory data Mean ± SD (median)	Control n = 20	SIRS n = 10	Septic shock-1/2 n = 20	Sepsis-3 n = 10	Septic shock-3 n = 10
mDO_2_bl (AU)	1346 ± 899 (1052)	706 ± 403 (786)^&^	635 ± 613 (347)^&^	916 ± 702 (900)	355 ± 355 (253)*
mDO_2_max (AU)	2392 ± 938 (2331)	1564 ± 639 (1562)^&^	1012 ± 731 (929)^&^	1326 ± 764 (1191)*	698 ± 569 (534)*
mVO_2_bl (AU)	388 ± 254 (318)	288 ± 173 (294)	206 ± 164 (147)^&^	263 ± 177 (243)	150 ± 133 (103)*
mVO_2_max (AU)	969 ± 369 (968)	677 ± 199 (696)^&^	490 ± 344 (353)^&^	603 ± 405 (482)*	377 ± 239 (337)*
MBFbl (AU)	191 ± 110 (172)	154 ± 87 (175)	121 ±110 (73)^&^	170 ± 121 (160)	71 ± 72 (53)*^,#^
MBFmax (AU)	301 ± 123 (298)	239 ± 88 (257)	159 ±121 (129)^&^	204 ± 134 (197)*	114 ± 94 (97)*
rHbbl (AU)	70.8 ± 10.2 (70.5)	47.8 ± 6.3 (48.0)^&^	56.1 ± 12.6 (55.0)^&,§^	55.0 ± 13.0 (57.0)*	57.2 ± 12.7 (52.1)*
rHbmax (AU)	87.3 ± 7.9 (90.2)	69.6 ± 8.6 (70.6)^&^	70.44 ± 12.6 (70.3)^&^	67.5 ± 15.5 (69.1)*	73.4 ± 8.6 (70.8)*
SaO_2_ /SpO_2_ (%)	95.8 ± 1.6 (96.0)	97.9 ± 1.8 (98.5)	92.9 ± 4.4 (93.5)^&,§^	94.1 ± 3.4 (94.0)	91.7 ± 5.1 (92.5)*
SvcO_2_bl (%)	66.9 ± 6.1 (68.0)	55.3 ± 8.6 (55.3)^&^	52.6 ± 16.5 (56.0)^&^	61.4 ± 7.8 (62.3)	43.7 ± 18.5 (45.9)*^,#^
SvcO_2_max (%)	79.2 ± 4.5 (78.7)	74.9 ± 12.1 (72.3)	65.2 ± 10.7 (65.5)^&,§^	72.5 ± 5.6 (72.6)*	57.8 ± 9.5 (61.1)*^,#^
SaO_2_ (%)—SvcO_2_bl (%)	28.8 ± 7.1 (27.5)	42.6 ± 8.0 (42.6)^&^	40.3 ± 15.3 (37.9)^&^	32.7 ± 9.8 (30.8)	48.0 ± 16.5 (43.4)*^,#^
SaO_2_ (%)—SvcO_2_max (%)	16.6 ± 5.2 (17.2)	23.0 ± 11.3 (25.0)	27.7 ± 10.0 (25.7)^&^	21.6 ± 6.7 (19.9)	33.9 ± 9.2 (33.1)*^,#^
mExO_2_bl (%)	29.8 ± 7.1 (28.6)	43.3 ± 8.3 (43.1)^&^	43.0 ± 17.2 (41.0)^&^	34.2 ± 9.1 (32.5)	51.9 ± 19.2 (46.9)*^,#^
mExO_2_max (%)	41.4 ± 7.5 (39.6)	46.7 ± 15.1 (46.0)	53.8 ± 15.6 (55.6)^&^	46.8 ± 14.1 (51.8)	60.8 ± 14.3 (63.0)*^,#^

**Figure 3:**
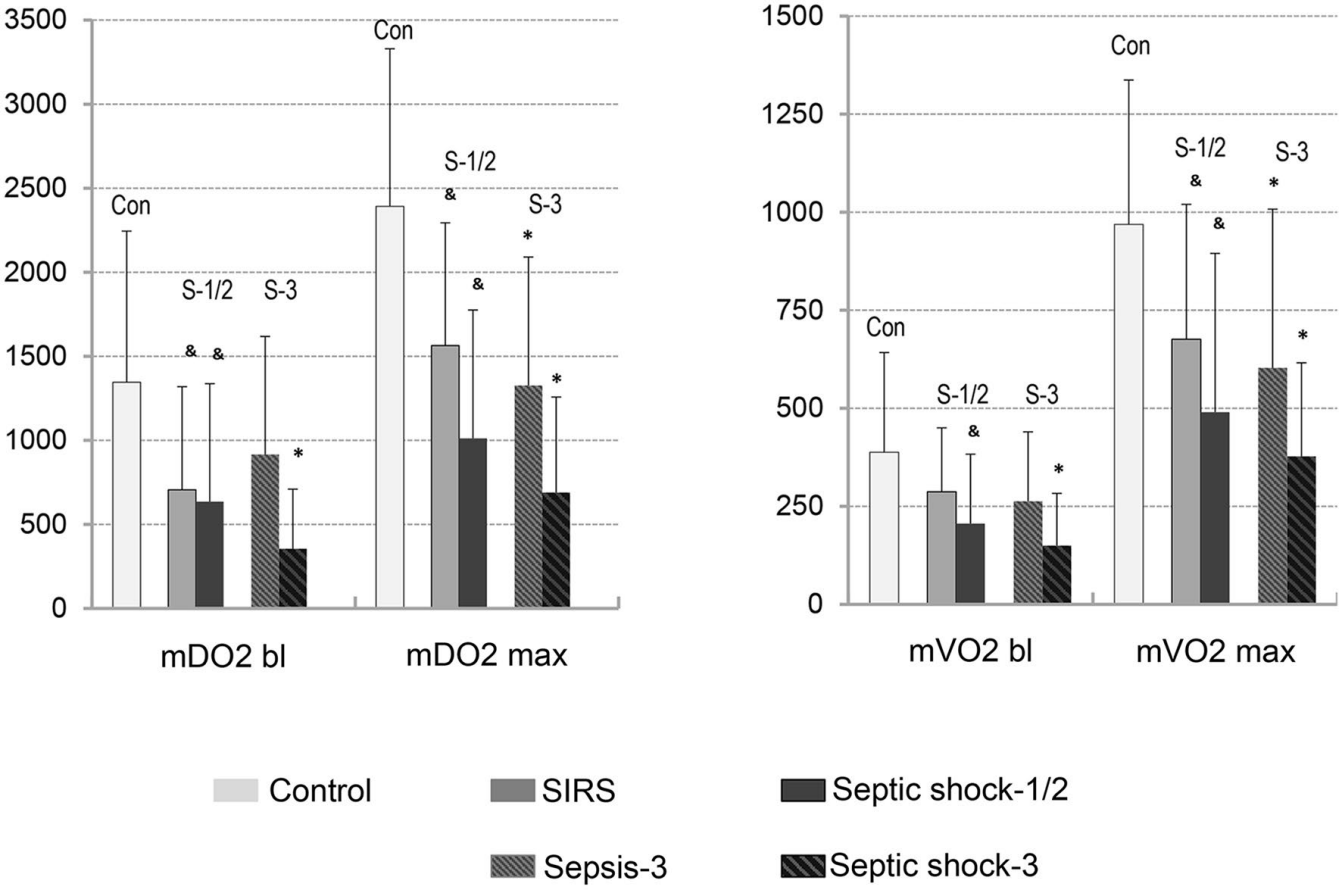
Skin microcirculatory oxygen delivery and uptake—intergroup comparisons; mDO2, microcirculatory oxygen delivery; mVO2, microcirculatory oxygen uptake; bl refers to baseline; max refers to maximum values determined upon reperfusion; data are presented as mean ± SD; & or * indicate “as compared to control”, respectively; a *p*-value < 0.05 was considered as statistically significant.

Values of microcirculatory oxygen delivery showed significant group differences between control and critically ill patient groups already at baseline, differences which were slightly more pronounced when maximum values after ischemia were analyzed (see also Fig. 3, left panel). Analyses of the individual factors determining baseline and maximum mDO2 revealed that oxygen delivery under inflammatory conditions was significantly diminished by both a decrease in MBF and arterial blood oxygen saturation with the lowest values in septic shock groups. However, flow and arterial blood oxygen saturation-limited microcirculatory oxygen delivery was partially compensated for by a less pronounced reduction in regional haemoglobin concentration (rHb) in septic shock-1/2 patients as compared with SIRS patients. As a result, differences in baseline and maximum mDO2 between SIRS and septic shock-1/2 patients did not reach level of significance. As compared with sepsis-3 patients, septic shock-3 patients showed lower values for mDO2 values under baseline and maximum conditions again due to lower values of arterial blood flow and oxygen saturation. However, flow and arterial blood oxygen saturation-limited microcirculatory oxygen delivery was again partially compensated for by a less pronounced reduction in regional haemoglobin concentration (rHb) in septic shock-3 patients as compared with sepsis-3 patients, leading to no significant differences in baseline and maximum mDO2 between sepsis-3 and septic shock-3 patients.

With respect to oxygen consumption, baseline and postischemic maximum values were decreased in SIRS and even more in septic shock patient groups as compared to control patients. However, the trend for lower baseline and maximum mVO_2_ in septic shock-1/2 patients compared to SIRS patients, alike mDO_2_, did not reach statistical significance (see also Fig. 3, right panel). As compared with sepsis-3 patients, baseline and maximum microcirculatory oxygen consumption rates were almost halved in sepsis-3 shock patients but the decrease did not reach the level of statistical significance.

The ratio of mVO_2_ to mDO_2_, i.e. microcirculatory oxygen extraction rate, determined under baseline conditions (mExO_2_bl) showed an increase for SIRS and septic shock groups as compared to control patients with no significant differences between SIRS and septic shock-1/2 patient groups. As compared to sepsis-3 patients, septic shock-3 patients showed significantly higher mExO_2_bl values (Table 2). Postischemic microcirculatory maximum oxygen extraction rate (mExO_2_max) increased up to an average value of 40% upon VOT already in control patients, with a tendency of higher values in SIRS and significantly higher values in septic shock patient groups as compared with control patients. In septic shock-3 patients mExO_2_max was highest and significantly increased as compared to sepsis-3 patients.

**Figure 4:**
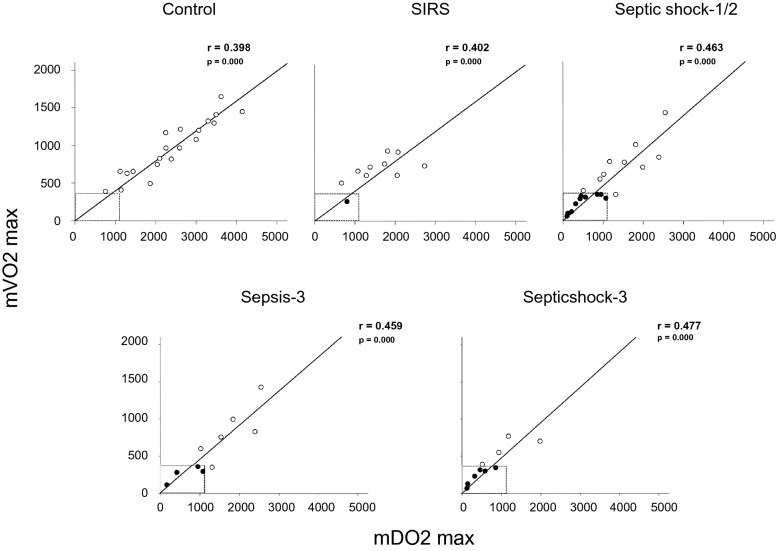
Scatter plots of microcirculatory maximum oxygen uptake and delivery values determined in control and critically ill patients; mVO2max, maximum microcirculatory oxygen uptake; mDO2max, maximum microcirculatory oxygen delivery; Scales of y—axes are the same for all plots. In all plots, a rectangle of the same size was placed with its lower left corner on the intersection of the abscissa and ordinate. The size of the rectangle was maximized in that its margins just reached the lowest mVO2max / mDO2max values for control patients, thereby defining an “unphysiological” region of mVO2max and mDO_2_max values. Values of maximal oxygen extraction rates are given by the slope calculated by pearson coefficient of correlation. For further explanation see text.

Plots of individual mVO2max by mDO2max values showed a linear relationship for all groups studied (Fig. 4). Of note, looking at dots of control patients a rectangle was positioned with the lower left corner on the intersection of the x-and y-axis with its size maximized not yet to reach any of the lowest individual mVO2max / mDO2max values. By doing so a region of mVO2max and mDO2max values is defined not observed in control patients. Accordingly, one may consider this region of mVO2max / mDO2max values an unphysiological one. When the same rectangle was applied to respective plots of the other patient groups, number and percentage of dots in the so defined region of “unphysiological” values increased with severity of illness.

Accordingly, none, i.e. 0 out of 20 individuals (0%) of control patients, 1 out of 10 individuals (10%) of SIRS patients, 10 out of 20 individuals (50%) of septic shock-1/2 patients, 4 out of 10 individuals (40%) of sepsis-3 patients and 6 out of 10 individuals (60%) of septic shock-3 patients exhibited mVO2max / mDO2max values within the rectangle. Worth to note, the order of correlation coefficient was: r(Control) = 0.398 < r(SIRS) = 0.402 < r(sepsis-3) = 0.459 < r(septic shock-1/2) = 0.463 < r(septic shock-3) = 0.477, suggesting a tendency for increased oxygen extraction with increasing inflammation and shock severity. As both mDO2 and mVO2 are strongly dependent on MBF and MBF might be dependent on cardiac output at the systemic level, cardiac index (CI) in septic patients (identified by sepsis-1/2 or sepsis-3 criteria) was tested for correlation to MBF, mDO2 and mVO2, respectively. As shown in Table 3, neither MBF nor mDO2 or mVO2 determined at baseline or reperfusional maximum correlated with CI with all variables determined simultaneously, making microcirculatory measurements of additional value going beyond information obtained by hemodynamic measurements at the macrocirculatory level. However, reperfusional maximum postcapillary venous oxygen saturation showed a trend for correlation with CI, an observation not explained by respective changes of the other microcirculatory parameters.

**Table 3:** Correlation between cardiac index and microcirculatory variables in septic patients; Septic patients consist of septic shock patients identified by sepsis-1/2 definition criteria comprising sepsis-3 and septic shock-3 patients identified by sepsis-3 definition criteria. MBF, microcirculatory blood flow; mDO2, microcirculatory oxygen delivery; mVO2, microcirculatory oxygen uptake; mExO2, microcirculatory oxygen extraction rate; SvcO2, postcapillary venous oxygen saturation; r, pearson correlation coefficient; *p* values of single tests are shown in parenthesis. Correlation coefficients reach level of significance below *p* = 0.01 according to Bonferroni correction for multiple testing.

Septic patients (n = 20)	MBF		mDO2		mVO2		mExO2		SvcO2	
	Baseline	Maximum	Baseline	Maximum	Baseline	Maximum	Baseline	Maximum	Baseline	Maximum
Cardiac Index	r = 0.355	r = 0.165	r = 0.067	r = 0.088	r = 0.081	r = 0.011	r = -0.431	r = -0.35	r = 0.45	r = 0.521
	(*p* = 0.14)	(*p* = 0.50)	(*p* = 0.78)	(*p* = 0.72)	(*p* = 0.742)	(*p* = 0.965)	(*p* = 0.065)	(*p* = 0.142)	(*p* = 0.053)	(*p* = 0.022)

**Figure 5:**
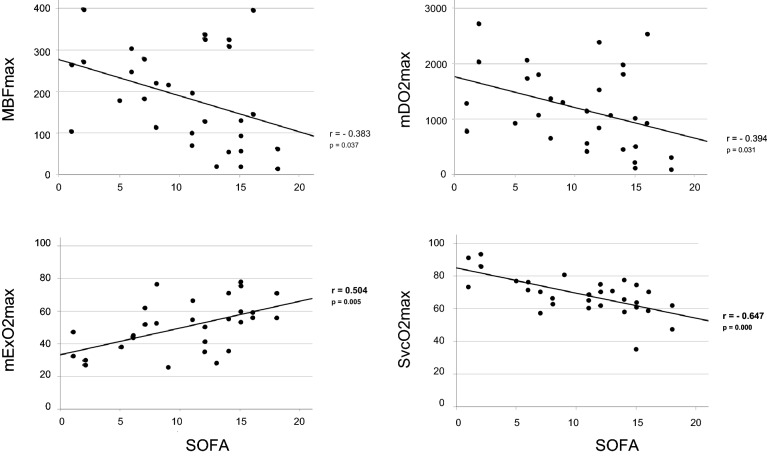
Scatter plots of microcirculatory variables and SOFA score determined in critically ill patients; Critically ill patients consist of patients suffering from SIRS and septic shock. Patients were identified by sepsis-1/2 definition criteria with septic shock-1/2 patients comprising sepsis-3 and septic shock-3 patients as defined by sepsis-3 definition criteria; MBFmax; maximum microcirculatory blood flow; mDO2max, maximum microcirculatory oxygen delivery; mExO2max, maximum microcirculatory oxygen extraction rate; SvcO2max, maximum postcapillary venous oxygen saturation; r, pearson correlation coefficient; Correlation coefficients reach level of significance below *p* = 0.0125 according to Bonferroni correction for multiple testing. Bold letters indicate significant *p*-values.

As the most important consequence of a restricted microcirculation is the occurrence of organ failure in a continuous spectrum of illness severity^26^, measured microcirculatory variables of all critically ill patients (SIRS and septic patients identified by either sepsis-1/2 or sepsis-3 criteria) were pooled and tested for correlation with the SOFA-, SAPS II—score and lactate plasma concentration. As shown in Table 4 SOFA-, SAPS II score and lactate plasma concentration were negatively but not significantly correlated with microcirculatory blood flow dependent oxygen delivery, while positively correlated with maximum microcirculatory oxygen extraction (mEx O_2_max). Consequently, the strongest correlation was observed for maximum postcapillary oxygen saturation (SvcO_2_max), which significantly decreased with increasing organ failure scores or lactate plasma concentration (Table 4). Data of post-ischemic measurements are also given by graphs in Fig. 5 showing strong and significant correlations of mExO_2_max and SvcO_2_max with the SOFA score. These findings are in agreement with an increasing compromise of the post-ischemic microcirculatory flow reserve with increasing illness severity. Testing for any other correlations between variables of the macrocirculation and variables of the microcirculatory level provided no additional significant information.

**Table 4:** Correlation between SOFA—, SAPS II- score, plasma lactate and microcirculatory variables in critically ill patients; Critically ill patients consist of patients suffering from SIRS and septic shock identified by sepsis-1/2 definition criteria with septic shock-1/2 patients comprising sepsis-3 and septic shock-3 patients identified by sepsis-3 definition criteria. SOFA, Sequential Organ Failure Assessment score; SAPS II, Simplified Acute Physiology II score; Lactate, lactate plasma concentration; MBF, microcirculatory blood flow; mDO2, microcirculatory oxygen uptake; mExO2, microcirculatory oxygen extraction rate; SvcO2, postcapillary venous oxygen saturation; r, pearson correlation coefficient; *p* values of single tests are shown in parenthesis. Correlation coefficients reach level of significance below *p* = 0.01 according to Bonferroni correction for multiple testing. Bold letters indicated significant *p*-values.

Critically ill patients (n = 30)	MBF		mDO_2_		mVO2		mExO_2_		SvcO_2_	
	Baseline	Maximum	Baseline	Maximum	Baseline	Maximum	Baseline	Maximum	Baseline	Maximum
SOFA	r = − 0184	r = − 0.383	r = − 0.074	r = − 0.394	r = − 0.199	r = − 0.180	r = 0.147	**r = 0.504**	r = − 0.260	**r = − 0.647**
	(*p* = 0.331)	(*p* = 0.037)	(*p* = 0.697)	(*p* = 0.031)	(*p* = 0.292)	(*p* = 0.342)	(*p* = 0.438)	(***p* = 0.005**)	(*p* = 0.166)	(***p* = 0.000**)
SAPS II	r = − 0.336	r = − 0.436	r = − 0.237	r = − 0.445	r = − 0.345	r = − 0.288	r = − 0.270	**r = 0.533**	r = – 0.377	**r = − 0.596**
	(*p* = 0.070)	(*p* = 0.016)	(*p* = 0.208)	(*p* = 0.014)	(*p* = 0.062)	(*p* = 0.122)	(*p* = 0.149)	(***p* = 0.002**)	(*p* = 0.040)	(***p* = 0.001**)
Lactate	r = − 0.354	r = − 0.376	r = − 0.346	r = − 0.439	r = − 0.262	r = − 0.241	**r = 0.542**	**r = 0.499**	**r = − 0.563**	**r = − 0.617**
	(*p* = 0.055)	(*p* = 0.040)	(*p* = 0.061)	(*p* = 0.015)	(*p* = 0.162)	(*p* = 0.199)	(***p* = 0.002**)	(***p* = 0.005**)	(***p* = 0.001**)	(***p* = 0.000**)

## Discussion

The aim of this work was to determine non-invasively maximum microcirculatory oxygen delivery and uptake using a non-invasive laser-doppler spectrophotometry system (O2C) at the bedside. Within the last two decades, developments and availability of clinically usable microcirculatory monitoring options produced growing knowledge about impaired microvascular function^27^. Interestingly, discrepancies with macrocirculatory parameters^28,29^ suggest no strict coupling between macro—and microcirculation as reported in the perioperative setting in the context of major surgery and in critical illness^5,28–32^. Some of the tools used in such studies directly visualise microvascular flow conditions by sidestream darkfield imaging (SDF), some focus on quantitative blood flow by laser doppler flowmetery (LDF), others examine tissue perfusion by carbon dioxide tonometry, or near infrared spectroscopy (NIRS)^20,33,34^. Although several very sophisticated techniques are nowadays available^5,29,33,35^, use of microcirculatory monitoring has not become daily routine at the ICU^29,36,37^. Moreover, end-points for microcirculation-focused resuscitation have still to be defined^29,36^ because of a lack of reliable parameters^38,39^.

The primary new approach of our study is to exploit the strengths of the used monitor (O2C)^12^. The O2C monitor was extensively investigated in many clinical settings (^40,41^) and sites (skin^17,19^, free lap^42^, cerebral^43^, buccal and gastric mucosa microperfusion^18,44^), and in combination with VOT to identify easy applicable global microcirculatory parameters which might be of potentially prognostic value and help guiding therapy.

### Limitations

In search of such techniques and parameters, one has to bear in mind several limitations. Well-known technical limitations of optical methods are artefacts induced by surrounding room light or patient movements. Microcirculatory impairments and heterogeneities in blood flow distribution are not directly visualised by saturation measurements and flux units are not available for laser-doppler data used in clinical settings^45^. Accordingly, previous control standard values have not been published so far. Our approach is evaluated based on differences in readouts for microcirculatory DO2 and VO2 between patient groups suffering from increasing severity of illness, ranging from otherwise healthy controls to septic shock patients. Critically ill patients were screened on a daily basis by sepsis-1/2 definition criteria, leading to identification of SIRS—and septic shock-1/2 patient groups. The septic shock-1/2 group was further classified by sepsis-3 criteria into sepsis-3 and septic shock-3 groups. Data obtained for septic shock-1/2 and septic shock-3 patients were not compared with each others as this was not the aim of the present study, which by the way was not empowered for this purpose. Despite of this, large epidemiological studies in the hospital setting comparing diagnoses of septic shock defined either by sepsis-1/2 or sepsis-3 criteria showed similar agreement (kappa = 0.412) between sepsis-1/2 and sepsis-3 shock, with a higher mortality in sepsis-3 shock patients than sepsis-1/2 shock patients^46^. These findings are in agreement with the rank order of estimated mortalities in our small sized study and with results reported by others for respective collectives in intensive care units^47^. Irrespective of the sepsis definition used to identify increasingly ill patient groups microcirculatory DO_2_ and VO_2_ decreased demonstrating incremental compromise of the microcirculation with increasing illness severity, findings in favor of proof of principle. This all the more as derangement of mDO2 and mVO2 occurred independently from cardiac output at the macrocirculatory level. Nonetheless, mDO2 and mVO2 are calculated values and therefore run the risk of being inaccurate. Another limitation of this study is that it was a single center one and had an observational design. Consequently, our approach can only give an incentive for further testing and comparison with other non-invasive and invasive techniques allowing insight into the microcirculation that is more precise. To this end, clinical parameters such as capillary refill time and the mottling score—although easy to perform—yield no quantitative data. Moreover, there are no publications to the best of our knowledge reporting on combination of capillary refill time or mottling score with vasoocclusive testing for assessment of microcirculatory postischemic flow reserve.

### Mathematical aspects

Here-applied equations for calculation of mDO2 and mVO2 are derived from their macrocirculatory counterparts. The systemic maximum oxygen uptake capacity, first defined by Hill and Lupon in 1923^48^, is limited in healthy individuals by the functionality of the cardiorespiratory system or oxygen carriers and essentially depends on maximum cardiac output, haemoglobin concentration and arterial oxygenation. It is expressed in millilitre per kilogram bodyweight and minute, calculated by unambiguous parameters—and clear weighting. By contrast MBF and rHb are dimensionless but yet quantitative variables detectable by laser-doppler flowmetry and white light spectrophotometry based on Doppler shift—and Lambert–Beer law^19^. As a result quantitative—although dimensionless—measurements of MBF and rHb, arterial and postcapillary venous oxygen saturation allow for calculation of oxygen delivery and consumption within and between individuals' microcirculation in a comparable fashion.

### Technical aspects

The used WLS system was validated in the pig brain in comparison to oxygen saturation of local venous blood^49^ and LDF is widely accepted as a reliable method of microcirculatory flow determination^23,29,45^. WLS is technically near to NIRS, which is meanwhile a standard method of tissue oximetry^21^, showed good reliability^19^ and allows quantitative detection of changes in tissue oxygen saturation^50^. LDF data correctly quantify relative flow changes and show tight correlations to those detected by other blood flow measurement techniques^18,51^. Site-dependency is a critical issue in microvascular function assessment^51,52^. Most commonly the volar forearm was used for VOT with NIRS or LDF^20,22,31,52–54^. Therefore the most frequently reported site, the thenar eminence^21^, was chosen.

VOT is a reliable functional test to detect overall changes in microvascular function with LDF and peak blood flow is not further elevated if occlusion time exceeds three minutes^52^. On the other hand, intermittend hypoxia leads to altered metabolic signals with long lasting effects^55^. That’s why rapidly repeated VOT is problematic and reproducibility of VOT is poor^19,51^ (intraindividual variability 12%^52^, coefficient of variation 9.2 ± 1.7%^19^). Additionally, marked variability of skin blood flow is obvious in the critically ill^56^. Nonetheless, clinical signs of compromised microcirculation were shown to be predictive for 14-day mortality in septic patients^8^.

### Findings

We provide first data of skin microcirculatory oxygen delivery and uptake demonstrating increasing compromise with respect to severity of underlying inflammatory disease. Specifically, as compared with control patients both baseline and post ischemic maximum mDO2 and mVO2 values were significantly decreased in patients with SIRS with this decrease even more pronounced in septic shock patients. The ischemic stimulus and subsequent reperfusion induce remarkable increases in mDO_2_ and mVO2. In fact, in control patients compared to baseline values at least twofold enhancement of mDO_2_ and mVO2 was recorded indicating an impressive post ischemic vascular flow reserve which obviously became more and more compromised by increasing disease severity. Interestingly, when the mVO2max/mDO2max ratio, i.e. the microcirculatory oxygen extraction ratio, was calculated, mean baseline values were at least 1.4 fold elevated in SIRS and in all septic patient groups as compared with control patients, most likely to compensate for limited oxygen delivery already under baseline conditions. As to be expected, microcirculatory oxygen extraction values further increased above baseline values in response to ischemia with start of reperfusion reaching maximum values in septic shock patients. When individual pairs of post ischemic maximum mDO_2_ and mVO2 values were plotted for each group, an interesting finding was made by placing a rectangle with the lower left corner on the intersection of the x-and y-axis. The size of this rectangle was defined in control patients in that its size was maximized not yet to reach any of the measured individual mVO2max / mDO2max values thus covering a region of mVO2max and mDO2max values not observed in control patients. As a result, this rectangle is very likely to represent an area of unphysiological mVO2max / mDO2max values obviously not observed in otherwise healthy patients without signs of inflammation. When the same rectangle was applied to respective plots of SIRS, sepsis and septic shock patients, number and percentage of mVO2max / mDO2max dots falling into this region of “unphysiological” values increased with severity of illness. One may speculate on the pathophysiological and –biochemical consequences of such microcirculatory oxygen delivery and consumption ratios in this unphysiological low value area for the outcome of SIRS and sepsis patients. If values in this newly identified low oxygen delivery / consumption value area really are of clinical relevance, it might be a useful tool for monitoring of a patients’ microcirculatory state and effectiveness of therapeutic measures. The possibility to enhance global DO2max and VO2max by multiple approaches is well described in sports science, where also a clear relationship to heart rate and thus flow exists^57^. Microcirculatory oxygen delivery and uptake depends on ‘functional capillary density’ (FCD), the number of capillaries along which red blood cells pass by in a given time^29^, i.e. velocity of flow and haemoglobin concentration and its oxygen saturation, summing up to the amount of oxygen delivered, followed by its transfer to mitochondrias as well as by its final utilisation^58^. Of note, the order of correlation coefficients of observed mVO2max/mDO2max ratios alike the mean maximal oxygen extraction values showed a tendency for increased oxygen extraction with increasing severity of inflammation likely to be indicative for an increasing imbalance in tissue’s oxygen need to delivery. The latter actually is hard to influence in fighting major causes of organ dysfunction and failure in trauma and sepsis, but FCD is recruitable in early stages of critical disease^5,59,60^ and promises improved outcome^61,62^ if it is a problem of convection. However, in doing so by fluid resuscitation, overinfusion is potentially fatal as it leads to edema and diffusion problems^28^ and thus should strictly be avoided^5^. The other factor, haemoglobin concentration, is also adjustable. There is some support for improved microcirculatory oxygen delivery by red blood cell (PRB) transfusion if microcirculatory flow is impaired^63,64^. However, concerns are growing about liberal transfusion strategies and recommendations for individualised decision-making are published^65^.

Taken together, monitoring of MBF, rHb, arterial and postcapillary venous oxygen saturation allow for calculation of mDO2max and mVO2max, which might serve as novel targets to guide therapy in adjusting volume and PRB transfusions to the needs of our patients at the microcirculatory level.

## Conclusion

We conclude that vasoocclusive testing with the combined laser-doppler spectrophotometry technique hold promise for assessment of skin microcirculatory oxygen delivery and uptake and its underlying factors. It is clinically applicable and has the potential to be a predictor of disease progression and to support physicians in guiding therapy. However, confirmatory studies have to be undertaken to test validity of our methodological approach and reproducibility of our results allowing for more specific guidance of therapy of critically ill patients at the microcirculatory level.

## Data Availability

The datasets analyzed during the current study are available from the corresponding author on reasonable request.
